# Zinc-mediated lysosomal activation by 1H10 enhances autophagy and attenuates tau pathology in Alzheimer’s disease models

**DOI:** 10.1186/s13041-026-01328-9

**Published:** 2026-07-08

**Authors:** Jae-Won Eom, Ki-Ryeong Kim, Dong-Hyuk Kim, Jae Gwang Song, Chaeeun Han, Hyung Wook Kim, Seoungjun Ha, Woon Ju Song, Yang-Hee Kim

**Affiliations:** 1 Department of Integrative Bioscience and Biotechnology, Sejong University, 209 Neungdong-ro, Gwangjin-gu, Seoul, 05006 Republic of Korea; 2 Zincure Corp, Seoul, 05006 Republic of Korea; 3 Institute of Bioscience and Biotechnology, Sejong University, Seoul, 05006 Republic of Korea; 4 Department of Chemistry, Seoul National University, Seoul, 08826 Republic of Korea

**Keywords:** Zinc, Lysosome, Autophagic flux, Tau, Alzheimer's disease

## Abstract

Impaired autophagic flux and lysosomal dysfunction contribute critically to the accumulation of pathological protein aggregates in Alzheimer’s disease (AD). Emerging evidence suggests that intracellular zinc dynamics regulate lysosomal function by modulating processes such as acidification and lysosomal biogenesis. We previously identified 1H10 as an AMP-activated protein kinase (AMPK) inhibitor and subsequently demonstrated its zinc-binding capacity and ability to regulate intracellular zinc homeostasis. Building on our prior findings that intra-lysosomal zinc promotes acidification and activates transcription factor EB (TFEB), we investigated whether 1H10 enhances lysosomal function through zinc mobilization in neurons, thereby improving autophagy and reducing pathological protein accumulation. In primary cortical neurons, 1H10 increased lysosomal abundance and enhanced lysosomal degradative capacity in a zinc-dependent manner, as demonstrated by increased cathepsin B activity and DQ-BSA degradation. It alleviated lysosomal dysfunction induced by v-ATPase inhibition and promoted autophagic flux, leading to reduced accumulation of amyloid-β (Aβ) and tau in neuronal models. In 5XFAD mice, 1H10 treatment showed trends toward improved spatial learning in the Morris water maze, reduced tau phosphorylation at Thr205 and Ser214, normalized LC3-II levels, and restored autophagic–lysosomal homeostasis, without significant changes in extracellular amyloid plaque burden. These findings indicate that zinc-mediated lysosomal activation by 1H10 enhances the autophagy-lysosomal pathway and attenuates tau pathology in AD models, suggesting that targeting lysosomal function may represent a potential therapeutic strategy for neurodegenerative disorders characterized by impaired proteostasis.

**Supplementary Information:**

The online version contains supplementary material available at https://doi.org/10.1186/s13041-026-01328-9.

## Introduction

Impaired lysosomal function and defective autophagic clearance are key contributors to the pathogenesis of neurodegenerative diseases, including Alzheimer’s disease (AD) [1–3]. Disruption of intracellular protein degradation pathways leads to the accumulation of aggregation-prone proteins such as amyloid-β (Aβ) and hyperphosphorylated tau, which are closely associated with neuronal dysfunction and cognitive decline [4–7]. Accordingly, strategies aimed at enhancing lysosomal function and restoring autophagic flux have emerged as promising therapeutic approaches to counteract proteinopathy and potentially slow disease progression [8–10].

Zinc ion (Zn^2+^), an essential trace element, plays critical roles in neuronal signaling, enzymatic activity, and cellular homeostasis in the brain [11, 12]. In addition to its well-established roles in synaptic transmission and plasticity [13, 14], zinc has recently been implicated in the regulation of autophagy and lysosomal function [15, 16]. Lysosomal zinc promotes acidification and activates transcription factor EB (TFEB), a master regulator of lysosomal biogenesis and autophagy-related gene expression [17]. These findings suggest that zinc homeostasis is critical for maintaining lysosomal integrity and degradative capacity in neurons.

Disruption of zinc homeostasis has been implicated in diverse neurological conditions. Excessive zinc accumulation contributes to neuronal injury in acute conditions such as ischemic stroke and traumatic brain injury [18–20], while in AD, zinc promotes Aβ aggregation and plaque formation [21, 22]. In contrast, reduced zinc availability has been reported during aging and in AD patients [23–26], which may impair lysosomal function and compromise protein clearance. These observations highlight a context-dependent role of zinc in neurodegeneration.

We previously identified 1H10 as a small-molecule inhibitor of AMP-activated protein kinase (AMPK) [27]. 1H10 protects neurons not only from zinc-induced neurotoxicity but also from glutamate-induced excitotoxicity, oxidative stress induced by H_2_O_2_ or Fe^2+^ exposure, and staurosporine-induced apoptosis. Moreover, intracerebroventricular administration of 1H10 conferred significant neuroprotection in a permanent middle cerebral artery occlusion (pMCAO) model of ischemic stroke [28]. Subsequent studies revealed that 1H10 binds zinc and modulates intracellular zinc distribution: in cortical neurons preloaded with zinc (300 µM, 15 min), treatment with 1H10 (10–40 µM) significantly reduced the intracellular zinc levels detected by FluoZin-3 (Kd for Zn^2+^ ≈ 9 nM [29]), and an in vitro competitive assay using Newport Green DCF (Kd for Zn^2+^ ≈ 1 µM [30, 31]) yielded an IC_50_ of 10.0 µM for zinc binding. Consistent with this activity, systemic administration of 1H10 was protective in an Experimental Autoimmune Encephalomyelitis (EAE) model of multiple sclerosis, in which 1H10 was interpreted as lowering pathologically elevated intracellular labile zinc [32]. Our subsequent observations, however, indicated that 1H10 can also act as a zinc shuttle, taking up extracellular zinc and releasing it intracellularly. Whether 1H10 ultimately depletes or supplements cytosolic free zinc is therefore determined not by the drug concentration per se, but by the prevailing intracellular zinc state and by the buffering capacity of intracellular zinc stores, principally the lysosome. This raised the unexplored possibility that, under conditions of low intracellular labile zinc, 1H10 might supplement intracellular zinc to support lysosomal function—a context particularly relevant to AD, in which lysosomal dysfunction is a central feature.

Here, we investigated the effects of 1H10 on lysosomal function, autophagy, and protein clearance in neuronal system and an AD mouse model. We show that 1H10 enhances lysosomal activity and promotes autophagic flux in a zinc-dependent manner in primary cortical neurons. Furthermore, chronic administration of 1H10 in 5XFAD mice reduces tau phosphorylation and shows modest improvement in spatial learning. These findings identify modulation of intracellular zinc dynamics as a mechanism by which 1H10 regulates lysosomal function and attenuates tau-related pathology.

## Materials and methods

### Competitive binding assay

The zinc-binding affinity of 1H10 was determined using a competitive binding assay with Mag-Fura-2 (AAT Bioquest, Pleasanton, CA, USA). A stock solution of 1H10 was diluted to a final concentration of 70 µM in 25 mM 3-(*N*-morpholino)propanesulfonic acid (MOPS, pH 7.0) buffer containing 2% (v/v) dimethyl sulfoxide (DMSO) in a quartz cuvette. Absorbance was recorded at 324 nm. This titration design—in which Zn^2+^ is titrated into a fixed [1H10 + Mag-Fura-2] mixture, rather than 1H10 titrated into a fixed [Zn^2+^ + Mag-Fura-2] mixture—was adopted to avoid concentration-dependent contributions of 1H10 absorbance at the 324 nm readout wavelength and to maintain a constant DMSO content (2%, v/v) throughout the titration. Mag-Fura-2 was then added to a final concentration of 12 µM, and absorbance was recorded again. Zinc titrations were performed by incrementally adding ZnCl_2_ (1.67 or 5 µM per step) to the Mag-Fura-2/1H10 mixture. After each addition, samples were incubated for 5 min to allow equilibration, followed by absorbance measurement. Absorbance values were corrected using the isosbestic point at 324 nm. As a control experiment, zinc titrations were also performed under the same buffer conditions using Mag-Fura-2 alone. Binding curves were generated by plotting the corrected absorbance values against the total concentration of added zinc, and the zinc-binding affinities of Mag-Fura-2 and 1H10 were determined by fitting the titration data to a 1:1 binding model using custom scripts implemented in DynaFit [33, 34]. To assess whether 1H10 measurably competed with Mag-Fura-2 for Zn^2+^, the titration data were additionally analyzed using both non-binding and 1:1 competition models in DynaFit. Model performance was evaluated by residual analysis.

## Cell cultures

Primary cerebrocortical neurons were obtained from embryonic day 14 (E14) ICR mice (ORIENT BIO, Gyeonggi-do, South Korea). Briefly, cerebral cortices were dissected from four embryos, mechanically dissociated, and plated onto poly-D-lysine (Merck Millipore, Burlington, MA, USA)-coated culture plates. Neurons were maintained in Dulbecco’s Modified Eagle’s Medium (DMEM; Invitrogen, Carlsbad, CA, USA) supplemented with 2 mM glutamine, 5% fetal bovine serum (FBS; Hyclone, Logan, UT, USA) and 5% horse serum (HS; Invitrogen). All experiments using primary neurons were performed at 10–12 days in vitro (DIV).

HEK293 cells and RGL-H4 cells (H4 glioma cells stably expressing tandem RFP-GFP-LC3) were maintained in high-glucose DMEM (Welgene, Gyeongsan, South Korea) supplemented with 10% FBS (Invitrogen) and 100 µg/ml gentamicin (Duchefa, Haarlem, Netherlands). Cells were maintained at 37 °C in a humidified atmosphere containing 5% CO_2_.

## Chemical treatments

For all cell-based experiment, the growth medium was replaced with Minimal Essential Medium (MEM; Invitrogen), and cells were pretreated with 1 µM 1H10 (InterBioScreen, Chernogolovka, Russia) for 30 min. Following pretreatment, cells were exposed to 100 nM bafilomycin A1 (Cayman, Ann Arbor, MI, USA) or 1 µM *N*,*N*,*N*′,*N*′-tetrakis(2-pyridylmethyl)ethylenediamine (TPEN, Merck Millipore) for the indicated time points at 37 °C in a humidified incubator containing 5% CO_2_. For western blot analysis of protein aggregates in HEK293 cells, 1H10 was applied at a concentration of 250 nM.

## Transient transfection

HEK293 cells were transiently transfected with 1 µg/ml pEGFP-C1-Aβ_1−42_ (Addgene, Cambridge, MA, USA) or Tau(P301L)-GFP plasmid using Lipofectamine 2000 (Invitrogen), according to the manufacturer’s instructions. Transfection was performed for 4 h, after which the medium was replaced with DMEM supplemented with 10% FBS and 100 µg/mL gentamicin. Cells were then incubated for an additional 24 h to allow protein expression, and experiments were conducted the following day. The Tau(P301L)-GFP plasmid was kindly provided by Dr. Jung-Jin Hwang (University of Ulsan College of Medicine, Seoul, Republic of Korea).

## Confocal imaging

For lysosomal zinc imaging, primary cerebrocortical neurons were cultured on 12-mm circular coverslips and incubated with 5 µM Zinpyr-1 (ZP-1, Cayman, Ann Arbor, MI, USA) and 75 nM LysoTracker Red DND-99 (LTR; Invitrogen) for 30 min at 37 °C. After removal of the dyes, cells were treated with the indicated compounds, fixed with 4% paraformaldehyde (PFA), washed twice with cold-PBS, mounted in fluorescence mounting medium and imaged within 24 h using a TCS SP5 confocal laser scanning microscope (Leica Microsystems, Wetzlar, Germany).

RGL-H4 cells were cultured on 18-mm circular coverslips, treated as indicated, fixed in 4% PFA, mounted in fluorescence mounting solution (Dako, Santa Clara, CA, USA). Images were acquired using the same confocal microscope. For quantification of autophagic vesicles, RFP-GFP-LC3-positive cells were randomly selected, and puncta were counted in at least five randomly chosen fields per condition.

### Microscopic detection of lysosomal activity and in situ cathepsin B enzymatic activity

Lysosomal activity was assessed by incubating primary cortical neurons with 75 nM LysoTracker Red (LTR) for 30 min at 37 °C. After washing with MEM, cells were treated with 1H10 and bafilomycin A1 as indicated. Fluorescence intensity was used as an indicator of lysosomal acidity.

In situ cathepsin B activity was evaluated using the Magic Red Cathepsin B Detection Kit (ImmunoChemistry Technologies, Minneapolis, MN, USA) according to the manufacturer’s instructions. Following 1H10 treatment, cells were incubated with 1X Magic Red substrate in MEM for 30 min at 37 °C. The cleaved fluorescent product, indicative of active cathepsin B, was then washed out with MEM. Fluorescence signals were detected in live cells using a fluorescence microscope (Thermo Fisher Scientific, Waltham, MA, USA), and quantified using ImageJ software (NIH, Bethesda, MD, USA). Representative images were randomly selected.

## DQ-BSA assay

DQ-BSA staining was performed on mouse cortical neurons cultured on 12-mm circular coverslips. Cells were pre-treated with the indicated drug for 30 min, after which the culture medium was replaced with medium containing 10 µg/ml DQ-BSA (Thermo Fisher Scientific, Waltham, MA, USA). Cells were then incubated at 37 °C for 4 h. Following incubation, the DQ-BSA-containing medium was removed, and cells were fixed with 4% paraformaldehyde (PFA) for 10 min at room temperature. Coverslips were washed twice with ice-cold phosphate-buffered saline (PBS) and mounted onto glass slides using a fluorescence mounting medium. Fluorescence imaging was performed within 24 h of mounting using a TCS SP5 confocal laser scanning microscope (Leica Microsystems, Wetzlar, Germany).

## Animals

5XFAD transgenic mice (B6.Cg-Tg(APPSwFlLon, PSEN1*M146L*L286V)6799Vas/Mmjax) were purchased from The Jackson Laboratory (Bar Harbor, ME, USA). Three-month-old female 5XFAD mice and age-matched wild-type littermates were randomly assigned to receive intraperitoneal injections of 1H10 (30 µg/kg) or vehicle (DMSO/5% hydroxypropyl-β-cyclodextrin (HPBCD)/saline [10:90 v/v]). The dose of 1H10 (30 µg/kg, i.p.) was selected based on prior in vitro efficacy observed in the micromolar range and the absence of definitive pharmacokinetic and blood-brain barrier permeability data. This dose was chosen to provide sustained systemic exposure and potential central nervous system target engagement during long-term treatment while minimizing the risk of overt toxicity. Treatment was administered five times per week for approximately six months (*n* = 20 per group). Behavioral testing, including the Morris water maze, was conducted after five months of treatment. Drug administration was maintained continuously throughout behavioral testing, and mice were sacrificed for biochemical and histological analysis approximately two weeks after completion of the Morris water maze (approximately 8.75 months of age), with the final injection given 1–2 days before sacrifice. This two-week interval was used to analyze the behavioral data and confirm that no additional behavioral testing was required before tissue collection; it did not represent a drug-free washout period. All animal procedures were approved by the Institutional Animal Care and Use Committee of Sejong University and were conducted in accordance with national guidelines of the care and use of laboratory animals.

### Morris water maze (MWM)

Spatial learning and memory were assessed using the Morris water maze. Mice were trained to locate a hidden platform in a circular pool (91 cm diameter, 45 cm height) filled with water maintained at 25 ± 1 °C. Training was conducted over six consecutive days, with four trials per day and randomized starting positions. Each trial ended when the mouse reached the platform or after a maximum of 60 s, at which point the mouse was guided to the platform.

On day 7, a probe trial was performed in which the platform was removed, and mice were allowed to swim freely for 60 s. Parameters analyzed included escape latency during training, time spent in each quadrant during the probe trial, and the number of crossings over the former platform location. Behavioral performance was recorded and analyzed using Any-maze software (Stoelting, Wood Dale, IL, USA). Animals exhibiting severe impairments in swimming ability were excluded from the analysis.

### Tissue preparation and Congo red staining

Mice were anesthetized with isoflurane and transcardially perfused with phosphate-buffered saline (PBS), followed by 4% paraformaldehyde (PFA). Brains were post-fixed in 4% PFA overnight and cryoprotected in 30% sucrose for 2–3 days. Samples were embedded in optimal cutting temperature (OCT) compound and frozen at -80 °C. Coronal brain sections (30 μm thickness) were prepared using a cryostat.

For amyloid plaque detection, mounted sections were stained with Congo red solution (0.5% Congo red in 50% ethanol) for 20 min, rinsed in tap water, and briefly differentiated in alkaline alcohol (1% NaOH in 50% ethanol). Sections were then rinsed, dehydrated through graded ethanol solutions (95% and 100%, 3 min each), and immediately examined under a fluorescence microscope (Thermo Fisher Scientific, Waltham, MA, USA). Congo red-positive plaques were quantified by counting stained deposits in defined brain regions.

### Immunohistochemistry

Brain sections were permeabilized with 0.2% Triton X-100 for 3–5 min and blocked with 1% bovine serum albumin (BSA; GenDEPOT, Barker, TX, USA) in PBS for 30 min at room temperature. Sections were incubated overnight at 4 °C with primary antibodies against phosphorylated tau (Thr205; Signalway Antibody LLC, Greenbelt, MD, USA) and total tau (Cusabio Technology LLC, Houston, TX, USA) in a humid chamber. After washing, sections were incubated with fluorescence-conjugated secondary antibodies for 2 h at room temperature, followed by nuclear staining with Hoechst-33258 (20 µg/ml; Invitrogen) for 15 min. Sections were mounted using fluorescence mounting medium (Dako) and imaged with a Leica TCS SP5 confocal laser scanning microscope (Leica Microsystems). Fluorescence intensity was quantified using ImageJ software (NIH, Bethesda, MD, USA).

### Western blot analysis

For total protein extraction, cells were lysed in RIPA lysis buffer (50 mM Tris; pH 7.5, 150 mM NaCl, 1% NP-40, 0.5% deoxycholic acid, 0.1% SDS, and 5 mM EDTA) supplemented with protease and phosphatase inhibitors (2 µg/ml aprotinin, 2 µg/ml leupeptin, 1 µg/ml pepstatin A, 1 mM PMSF, 1 mM sodium orthovanadate, 5 mM sodium fluoride, and 10 mM sodium pyrophosphate). For detection of protein aggregates, HEK293 cells were lysed using a mild lysis buffer (20 mM HEPES pH 7.4, 150 mM NaCl, 1% Triton X-100, 1 mM EDTA) containing the same inhibitor cocktail. Lysates were centrifuged at 10,000 rpm for 5 min at 4 °C, and supernatants were subjected to SDS-PAGE. Mouse brain cortices were homogenized in RIPA buffer and centrifuged at 13,200 rpm for 20 min at 4 °C. Supernatants were collected and resolved by SDS-PAGE. Primary antibodies included anti-p62 (MBL, Nagoya, Japan), anti-LC3 (Novus, Littleton, CO, USA), anti-GFP (Santa Cruz Biotechnologies, Dallas, TX, USA), anti-Aβ (6E10; Biolegend, San Diego, CA, USA), anti-phospho-tau (Ser404, Cusabio; Ser214 and Thr205, Signalway Antibody), anti-tau (Cusabio), anti-pro-cathepsin B (CTSB; Abcam, Cambridge, UK), anti-mature CTSB (Cell signaling Technology, Danvers, MA, USA), anti-cathepsin D (CTSD; Abcam), and anti-actin (Abbkine, China). Actin was used as the loading control. HRP-conjugated secondary antibodies and Enhanced ChemiLuminescence (ECL) reagents (Abbkine) were used for signal detection. Band intensity was captured using a gel documentation system (BIS 303 PC, Bio-Imaging Systems, Israel) and quantified using ImageJ software.

### Statistical analysis

All data are presented as mean ± standard error of the mean (SEM). Comparisons among multiple groups were performed using one-way analysis of variance (ANOVA), followed by Dunnett’s or Tukey’s post-hoc test, as appropriate. Comparisons between two groups were analyzed using two-tailed Student’s *t*-tests. Exact *p* values and statistical details are provided in the figure legends. Statistical significance was defined as *p* < 0.05; “ns” denotes no significant difference. All statistical analyses were conducted using GraphPad Prism software (GraphPad Software, San Diego, CA, USA).

## Results

### 1H10, a zinc-binding small molecule, increases lysosomal zinc levels and enhances lysosomal activity

**Fig. 1:**
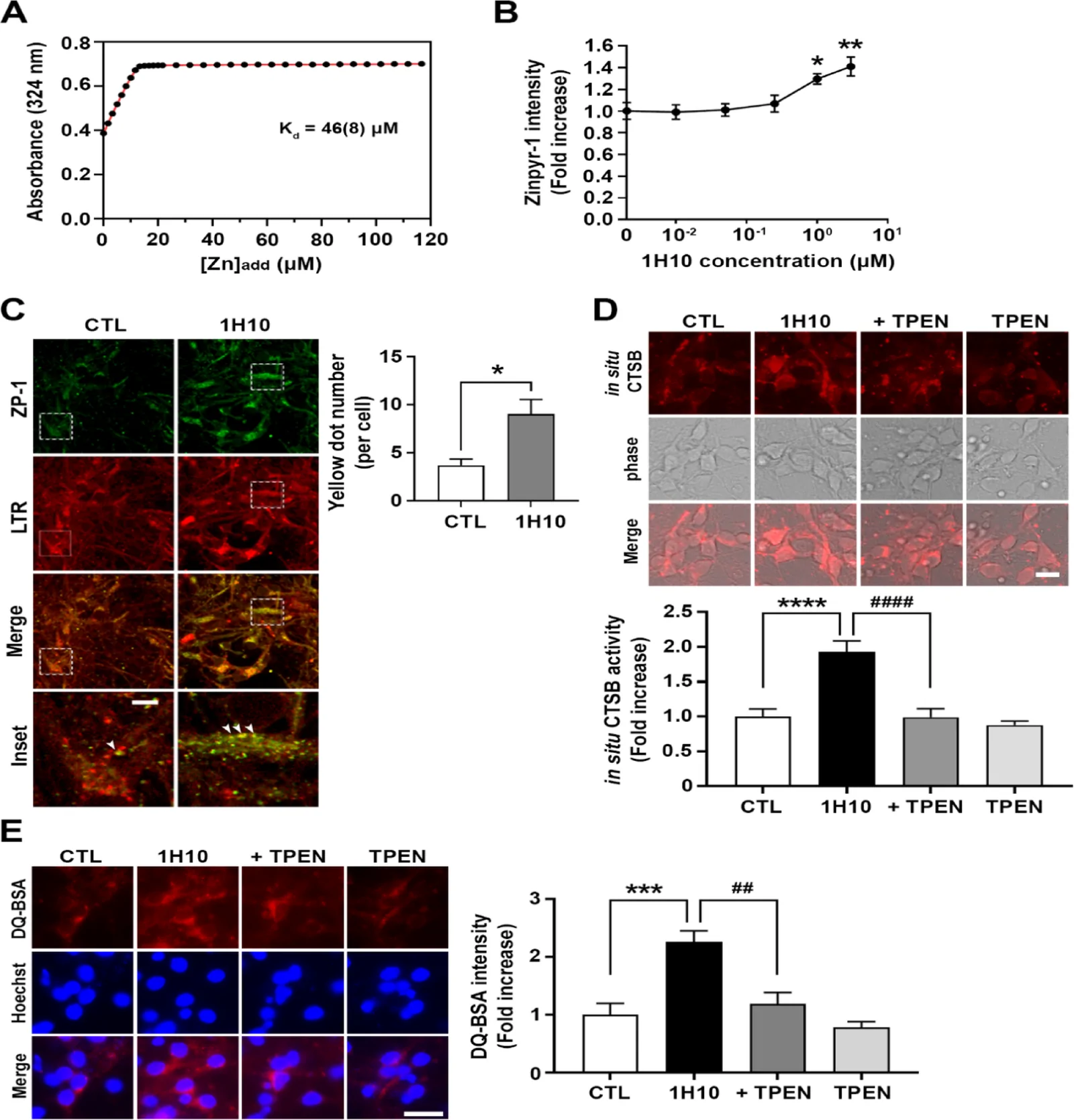
1H10 elevates lysosomal labile zinc levels and enhances lysosomal protease activity in mouse cortical neurons. **A** Zinc-binding property of 1H10. A competitive zinc binding assay was performed using Mag-Fura-2. Absorption at 324 nm was measured following stepwise titration of ZnCl_2_ into a solution containing 70 µM 1H10 and 12 µM Mag-Fura-2. The calculated dissociation constant (K_d_) of 1H10 for zinc was approximately 46 µM. **B** Quantification of intracellular labile zinc levels. Primary cortical neurons were treated with increasing concentrations of 1H10 (0, 0.01, 0.05, 0.25, 1 or 3 µM) for 1 h and stained with Zinpyr-1 (ZP-1). Fluorescence intensity was quantified using ImageJ (mean ± SEM, *n* = 7 fields from ≥ 3 independent experiments). *p < 0.05, or **p < 0.01 versus 0 µM 1H10 by one-way ANOVA followed by Dunnett’s post-hoc test. **C** Confocal images and quantification of ZP-1 and LysoTracker Red (LTR) co-staining. Neurons treated with 1 µM 1H10 for 1 h exhibited increased LTR-positive puncta, indicative of acidic lysosomes, along with enhanced ZP-1 fluorescence. Merged images show a significant increase in colocalized puncta (yellow; arrowheads), representing zinc-enriched lysosomes. Scale bar, 5 μm. Insets show magnified views of boxed regions. The bar graph indicates the number of colocalized puncta per cell (mean ± SEM, *n* = 3). *p < 0.05 by two-tailed Student’s *t*-test. **D** Cathepsin B (CTSB) activity. In situ CTSB activity was measured 2 h after treatment with vehicle (CTL), 1 µM 1H10, 1 µM 1H10 plus 1 µM TPEN, or TPEN alone. Representative fluorescence images (top) and quantitative analysis (bottom) are shown. Scale bar, 20 μm. Data represent fluorescence intensity as a measure of CTSB activity (mean ± SEM, *n* = 9 different fields taken from ≥ 4 independent experiments). ****p < 0.0001 versus sham wash control; ^####^*p* < 0.0001 versus 1H10 by one-way ANOVA with Tukey’s post-hoc test. **E** Bulk lysosomal proteolytic activity assessed by DQ-BSA dequenching. Primary cortical neurons were treated with vehicle (CTL), 1 µM 1H10, 1 µM 1H10 plus 1 µM TPEN, or TPEN alone, and DQ-BSA fluorescence was measured as a readout of bulk lysosomal proteolytic activity. Representative images (top) and quantitative analysis (bottom) are shown. Scale bar, 20 μm. Data represent DQ-BSA fluorescence intensity (mean ± SEM, *n* = 5 fields from ≥ 3 independent experiments). ***p < 0.001 versus sham wash control; ^##^*p* < 0.01 versus 1H10 by one-way ANOVA with Tukey’s post-hoc test. 1H10 significantly increased DQ-BSA fluorescence relative to CTL, and this increase was abolished by co-treatment with TPEN, whereas TPEN alone had no effect

Our previous study demonstrated that 1H10 binds zinc with an IC_50_ of ~ 10 µM, as determined using the zinc-sensitive fluorescent probe Newport Green DCF [32]. To further characterize the zinc-binding properties of 1H10, we measured its dissociation constant (K_d_) using a competitive binding assay with Mag-Fura-2. The calculated K_d_ was approximately 46 µM (Fig. 1A), confirming the zinc-binding capacity of 1H10.

To determine whether 1H10 alters intracellular zinc levels in neurons, primary cortical neurons were treated with increasing concentrations of 1H10. Quantitative analysis of Zinpyr-1 fluorescence revealed a significant increase in labile zinc levels at ≥ 1 µM (Fig. 1B). Consistent with these findings, fluorescence imaging showed that treatment with 1 µM 1H10 markedly enhanced Zinpyr-1 fluorescence, indicating elevated intracellular labile zinc levels (Fig. 1C).

We next examined the effects of 1H10 on lysosomal zinc accumulation. Neurons were co-stained with Zinpyr-1 and LysoTracker Red (LTR), a probe that labels acidic organelles. Following 1 h of 1H10 treatment, the number of colocalized (yellow) puncta—representing labile zinc within acidic lysosomal compartments—increased more than twofold compared with controls, indicating enhanced zinc accumulation in lysosomes (Fig. 1C).

We then assessed whether increased lysosomal zinc was associated with enhanced lysosomal function. In situ cathepsin B activity was significantly increased in neurons treated with 1H10, and this effect was markedly attenuated by the zinc chelator TPEN (*N*,*N*,*N*′,*N*′-tetrakis(2-pyridylmethyl)ethylenediamine), supporting a zinc-dependent mechanism underlying the enhancement of lysosomal proteolytic activity (Fig. 1D). Similarly, bulk lysosomal proteolytic activity, assessed by dequenching of DQ-BSA, was significantly increased following 1H10 treatment. This effect was abolished by co-treatment with TPEN, whereas TPEN alone had no significant effect (Fig. 1E). Together, these data indicate that 1H10 enhances overall lysosomal degradative capacity in a zinc-dependent manner.

These findings demonstrate that 1H10 elevates intracellular, particularly intralysosomal, zinc levels and lysosomal proteolytic activity in neurons.

### 1H10 partially restores autophagic flux and promotes degradation of Aβ and mutant tau

Autophagic flux requires coordinated autophagosome formation and subsequent fusion with lysosomes to enable degradation of sequestered cargo [35, 36]. Given that 1H10 enhances lysosomal function, we examined whether it could restore autophagic flux under conditions of lysosomal dysfunction induced by bafilomycin A1 (BafA1), a v-ATPase inhibitor that elevates lysosomal pH and disrupts autophagosome–lysosome fusion [37, 38].

**Fig. 2:**
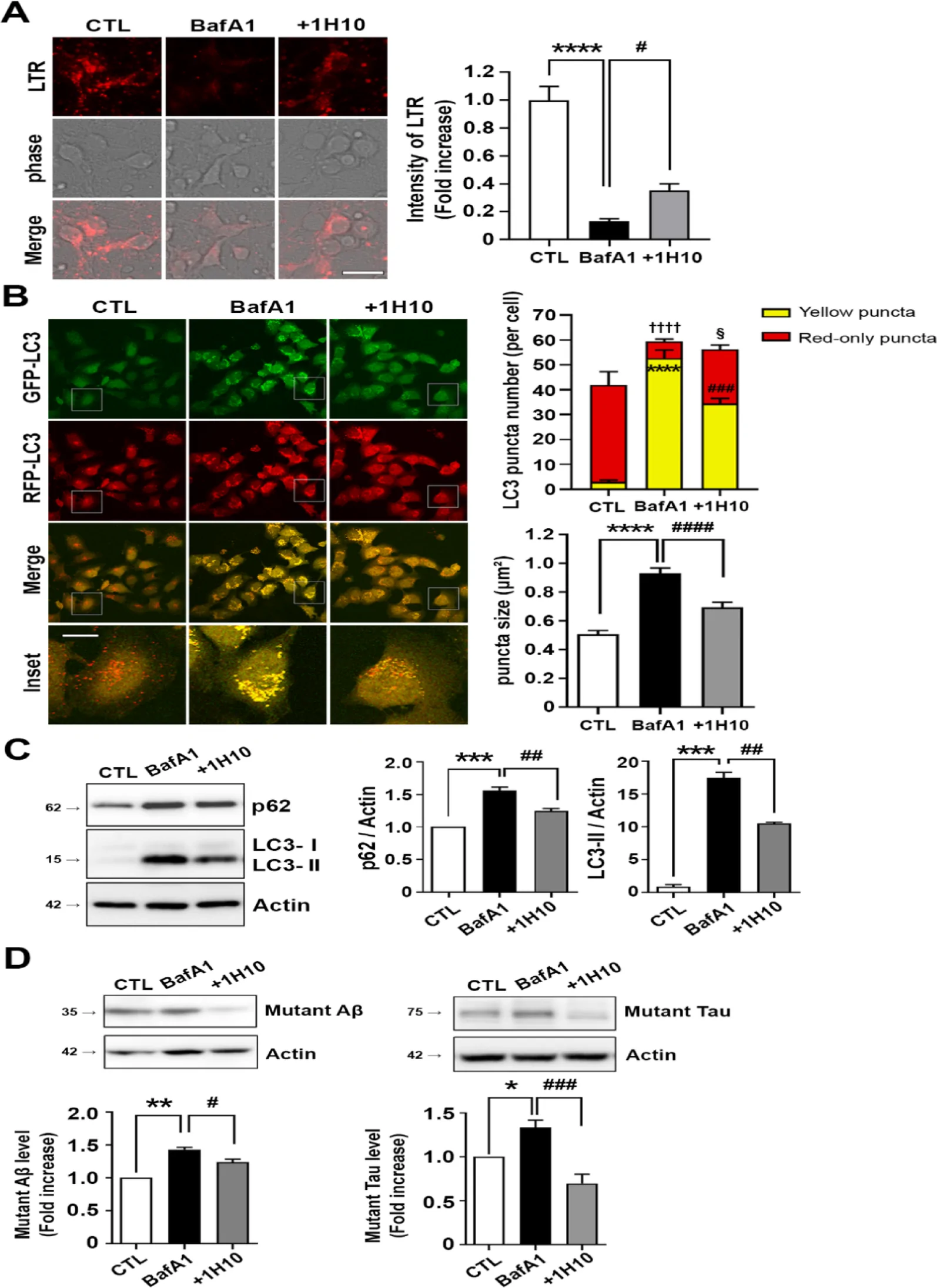
1H10 restores autophagic flux and reduces the accumulation of AD-associated protein aggregates. **A** Effect on lysosomal acidification. Primary cortical neurons were treated for 15 min with vehicle (CTL), 100 nM bafilomycin A1 (BafA1), or BafA1 plus 1 µM 1H10. BafA1 markedly reduced LTR fluorescence, indicating lysosomal alkalinization, whereas co-treatment with 1H10 significantly restored LTR intensity. Representative images (left) and quantitative analysis (right) are shown. Scale bar, 20 μm. Fluorescence intensity was quantified using ImageJ (mean ± SEM, *n* = 14 different fields taken from ≥ 3 independent experiments). ****p < 0.0001 versus CTL; ^#^*p* < 0.05 versus BafA1 by one-way ANOVA with Tukey’s post-hoc test. **B** Effect of 1H10 on autophagic flux in RFP-GFP-LC3-expressing H4 (RGL-H4) cells. Cells were incubated for 12 h under low-serum conditions in the presence of vehicle, BafA1, or BafA1 plus 1 µM 1H10. BafA1 treatment resulted in the accumulation of large yellow puncta (RFP⁺/GFP⁺ autophagosomes), reflecting impaired autophagosome–lysosome fusion. Co-treatment with 1H10 reduced GFP-positive puncta and increased red-only puncta, consistent with partial restoration of autophagic flux. Representative confocal images (left) and quantitative analysis of puncta number and size (right) are shown. Scale bar, 10 μm. Data are presented as mean ± SEM (*n* = 6). ****p < 0.0001 (CTL vs. BafA1, yellow puncta number and size); ^###^*p* < 0.001, ^####^*p* < 0.0001 (BafA1 vs. BafA1 + 1H10, yellow puncta number and size); ^††††^*p* < 0.0001 (CTL vs. BafA1, red-only puncta); ^§^*p* < 0.05 (BafA1 vs. BafA1 + 1H10, red-only puncta) by one-way ANOVA with Tukey’s post-hoc test. **C** Western blot analysis of autophagy-related markers. Primary cortical neurons were treated with BafA1 alone or BafA1 plus 1 µM 1H10 for 12 h, followed by analysis of p62 and LC3 expression. BafA1 induced the accumulation of p62 and LC3-II, both of which were reduced by co-treatment with 1H10. Representative immunoblots are shown together with quantitative analysis of p62 and LC3-II normalized to actin (mean ± SEM, *n* = 3). ***p < 0.001 (CTL vs. BafA1); ^##^*p* < 0.01 (BafA1 vs. BafA1 + 1H10) by ANOVA with Tukey’s post-hoc test. **D** Clearance of aggregation-prone proteins. HEK293 cells transiently expressing pEGFP-C1-Aβ_1−42_ or Tau(P301L)-GFP were treated with BafA1 alone or BafA1 plus 250 nM 1H10 for 6 h (Aβ) or 12 h (Tau). BafA1 markedly increased the accumulation of Aβ and mutant tau, whereas co-treatment with 1H10 significantly reduced their levels. Bar graphs show quantitative analysis (mean ± SEM, *n* = 2–3). *p < 0.05, **p < 0.01 versus CTL; #*p* < 0.05, ^###^*p* < 0.001 versus BafA1 by one-way ANOVA with Tukey’s post-hoc test

Treatment with BafA1 markedly reduced LTR fluorescence, consistent with lysosomal alkalinization and impaired lysosomal function. Co-treatment with 1H10 significantly increased LTR intensity, suggesting a partial recovery of lysosomal acidity and activity (Fig. 2A). To further assess autophagic flux, we utilized RFP–GFP–LC3–expressing H4 astroglioma cells (RGL-H4). Under autophagy-inducing low-serum conditions, control cells exhibited an increase in red-only puncta, corresponding to autolysosomes in which GFP fluorescence is quenched in acidic compartments. In contrast, BafA1-treated cells accumulated enlarged yellow puncta (RFP⁺/GFP⁺), indicative of impaired autophagosome–lysosome fusion and defective acidification. Co-treatment with 1H10 increased the proportion of red-only puncta and reduced the size of LC3-positive structures, consistent with a partial restoration of autophagic flux despite persistent v-ATPase inhibition (Fig. 2B).

To corroborate these observations, autophagy-related markers were analyzed by Western blotting. BafA1 treatment resulted in pronounced accumulation of p62 and LC3-II, reflecting impaired autophagic degradation. Co-treatment with 1H10 significantly reduced p62 levels and LC3-II accumulation induced by BafA1 (Fig. 2C), consistent with enhanced autophagic degradation.

Finally, we examined whether 1H10 influences the accumulation of aggregation-prone proteins. In HEK293 cells expressing Aβ_1−42_–GFP or mutant Tau(P301L)–GFP, BafA1 induced substantial accumulation of both proteins. Co-treatment with 1H10 markedly reduced their levels (Fig. 2D), indicating enhanced clearance under conditions of impaired lysosomal function.

These findings indicate that 1H10 promotes autophagic flux and facilitates the degradation of aggregation-prone proteins, including Aβ and mutant tau, under conditions of lysosomal stress.

### 1H10 administration is associated with modest, non-significant trends toward improved spatial learning and memory performance in 5XFAD mice

**Fig. 3:**
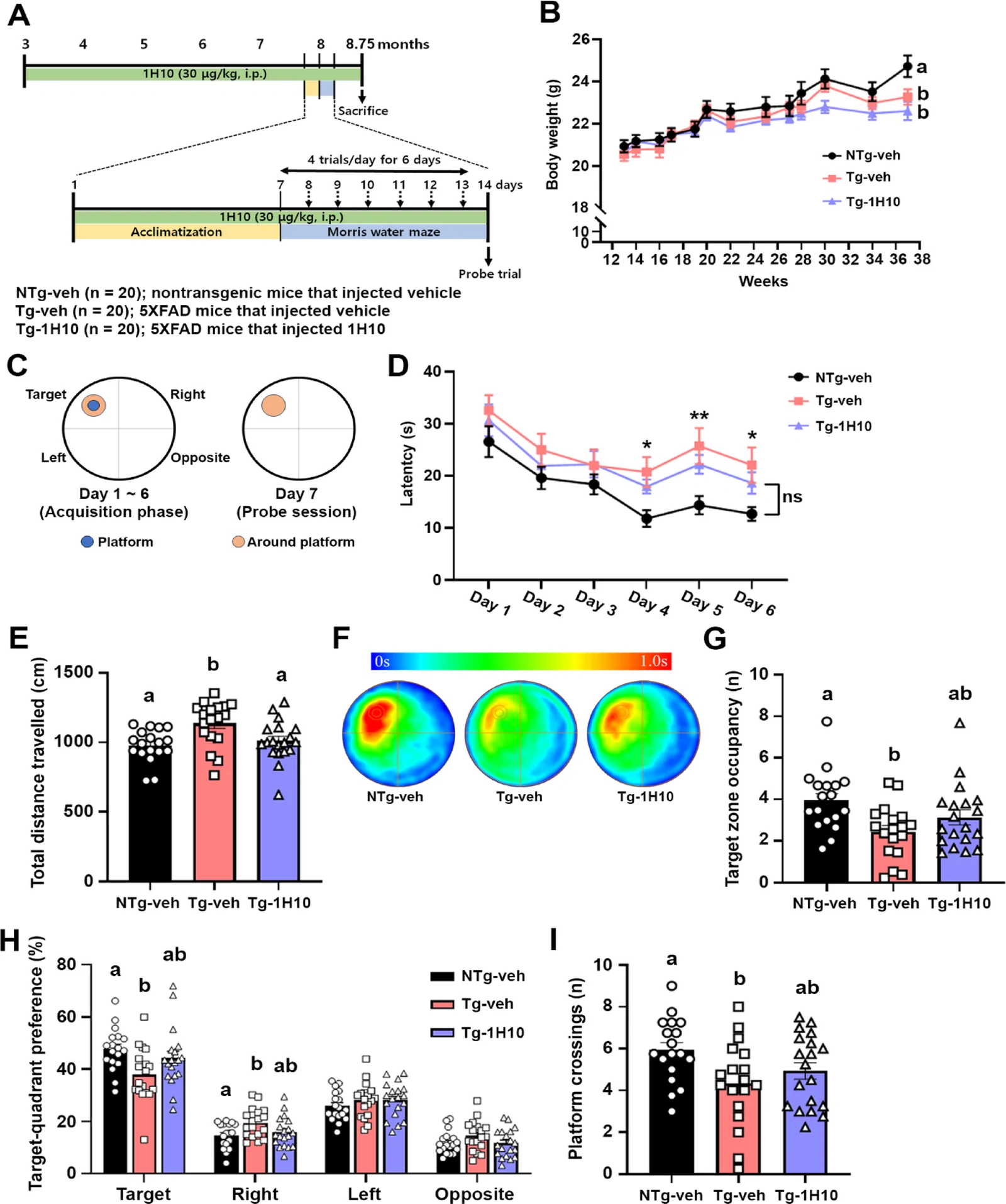
Effects of 1H10 on spatial learning and memory performance in 5XFAD mice. **A** Experimental timeline of the Morris water maze (MWM) test. **B** Weekly monitoring of body weight throughout the experimental period. **C** Schematic illustration of the MWM pool used for behavioral assessment, indicating the location of the hidden escape platform during acquisition training and the quadrant designation and target zone (platform radius + 6 cm) used for the probe-session analysis. **D** Escape latency to locate the hidden platform during six consecutive days of acquisition training. NTg-veh mice showed progressively reduced escape latencies, whereas Tg-veh mice exhibited prolonged latencies from day 4 onward. Escape latencies of Tg-1H10 mice did not differ significantly from those of either NTg-veh or Tg-veh mice across training days. **E** Total distance traveled during the probe session. **F** Representative heatmaps showing spatial occupancy during the probe session. **G** Target-zone occupancy (time spent in the target zone surrounding the former platform location; platform radius + 6 cm) during the probe session. **H** Target-quadrant preference (time spent in each quadrant) during the probe session. **I** Platform crossings (number of crossings over the former platform location) during the probe session. Data are presented as mean ± SEM (Fig. 3B: *n* = 20 per group; Fig. 3D and probe-trial parameters **E** and **G**–**I**: NTg-veh, *n* = 18; Tg-veh, *n* = 19; Tg-1H10, *n* = 18, reflecting post-hoc exclusion of one to two animals per group that did not display reliable swimming behavior). Statistical analyses for escape latency during acquisition training (**D**) were performed using one-way ANOVA followed by Dunnett’s post-hoc test, with NTg-veh as the reference group. Body weight data (**B**) were analyzed by two-way ANOVA, whereas probe-trial parameters (E, G–I) were analyzed by one-way ANOVA followed by Tukey’s post-hoc test. Groups not sharing a common letter are significantly different (*p* < 0.05); for example, groups labeled “a” and “b” differ significantly, whereas a group labeled “ab” does not differ significantly from either “a” or “b”. An asterisk (*) denotes *p* < 0.05 and a double asterisk (**) denotes *p* < 0.01, specifically for comparisons between NTg-veh and Tg-veh in Fig. 3D based on Dunnett’s post-hoc test. “ns” denotes no significant difference. Exact p-values are provided in the source data

To assess the in vivo therapeutic potential of 1H10, the compound was administered intraperitoneally to 5XFAD transgenic (Tg) mice for approximately six months, from 3 to 8.75 months of age. Spatial learning and memory were subsequently evaluated using the Morris water maze (MWM) (Fig. 3A). Consistent with previous reports, vehicle-treated 5XFAD transgenic mice (Tg-veh) exhibited significantly lower body weights than vehicle-treated non-transgenic littermates (NTg-veh) [39–41]. Body weights were comparable between 1H10-treated and vehicle-treated Tg mice (Tg-1H10 and Tg-veh), indicating that long-term administration of 1H10 did not affect body weight in 5XFAD mice (Fig. 3B).

The MWM pool layout, including the position of the hidden escape platform, the quadrant designation used for the probe-session analysis, and the target zone surrounding the former platform location, is schematized in Fig. 3C. During the acquisition phase of the MWM, NTg-veh mice progressively reduced their latency to locate the hidden platform across training days, whereas Tg-veh mice maintained prolonged escape latencies, indicative of impaired spatial learning. A significant difference between NTg-veh and Tg-veh mice emerged on day 4 and persisted through days 5 and 6. Tg-1H10 mice exhibited escape latencies that were modestly lower than those of Tg-veh mice during the later training days, while remaining clearly higher than those of NTg-veh controls (Fig. 3D). Although these differences did not reach statistical significance at any individual time point, Tg-1H10 mice consistently showed lower escape latencies than Tg-veh mice across the late training phase, indicating a directional trend toward improved performance during acquisition (Fig. 3D).

In the probe trial, Tg-veh mice exhibited significantly increased total swim distance compared with NTg-veh controls, indicative of inefficient search behavior. Notably, chronic 1H10 administration significantly reduced swim distance to levels comparable to NTg-veh mice, indicating improved search efficiency (Fig. 3E). Consistent with this finding, qualitative heatmap analysis revealed a partially refined search pattern in Tg-1H10 mice compared with Tg-veh mice, albeit less focused than in NTg-veh controls (Fig. 3F). In contrast, classical spatial memory indices showed more limited effects. Tg-veh mice displayed significant impairments in target-zone occupancy, target-quadrant preference, and platform crossings relative to NTg-veh controls. Although Tg-1H10 mice consistently exhibited intermediate or NTg-like trends across these measures, none reached statistical significance (Fig. 3G, H, I).

Together, these results indicate that while 1H10 does not significantly improve classical spatial memory indices, it enhances search efficiency and promotes a modest shift toward improved spatial search behavior in 5XFAD mice.

### 1H10 reduces tau phosphorylation and alters autophagy–lysosomal markers independently of amyloid pathology in 5XFAD mice

Following completion of behavioral testing at 8 months of age, and while still receiving continuous 1H10 treatment, 5XFAD mice were sacrificed approximately two weeks after the Morris water maze for biochemical and histological analyses. We examined pathological markers related to protein aggregation and autophagy-lysosomal function, including Aβ, senile plaques, phosphorylated tau, autophagy-related proteins (LC3 and p62), and lysosomal proteases (cathepsin B and D).

**Fig. 4:**
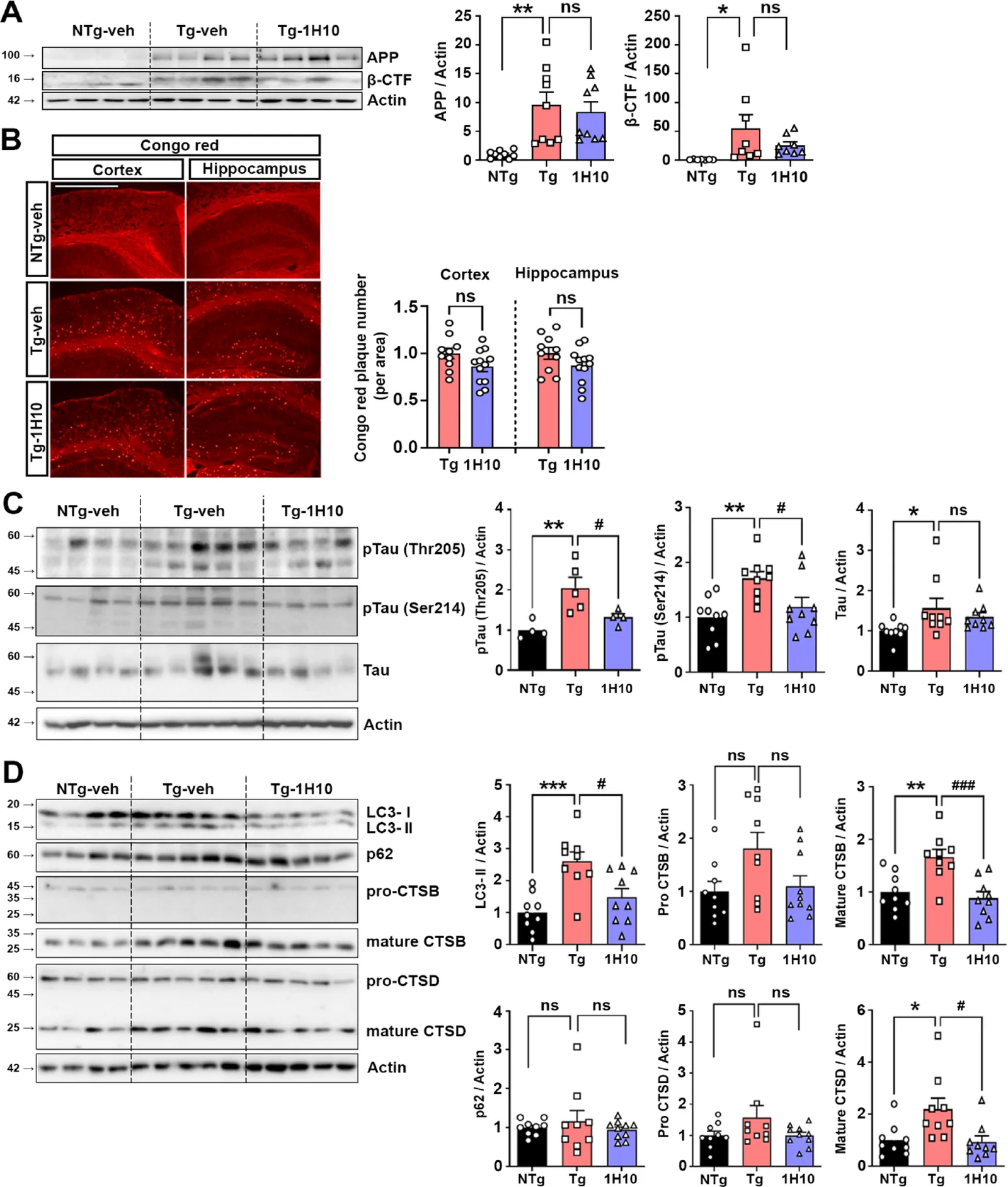
1H10 selectively reduces phosphorylated tau levels without altering amyloid pathology in 5XFAD mice. **A** Western blot analysis of amyloid-related species in cerebral cortical lysates using the 6E10 antibody, which resolves full-length APP (~ 100 kDa) and the β-secretase–cleaved C-terminal fragment (β-CTF/C99; ~16 kDa) on the same membrane. Molecular weight markers (kDa) are indicated to the left of the blot. Quantification of APP and β-CTF band intensities (normalized to Actin) is shown as mean ± SEM (*n* = 9). Both species were significantly increased in Tg-veh mice compared with NTg-veh controls; chronic 1H10 treatment produced a modest, non-significant decreasing trend in β-CTF (*p* > 0.05, Tg-veh vs. Tg-1H10). **B** Representative Congo red staining of amyloid plaques in the cortex and hippocampus. Scale bar, 1000 μm. Quantification of Congo red-positive plaque burden is shown as mean ± SEM (*n* = 10–12), with no significant difference (ns) between Tg-veh and Tg-1H10 mice. **C** Western blot analysis of phosphorylated tau (Thr205 and Ser214) and total tau in the cerebral cortical lysates. Quantification is shown as mean ± SEM (*n* = 4–9). Phosphorylated tau levels and total tau levels were quantified after normalization to Actin. **D** Western blot analysis of autophagy- and lysosome-related proteins, including LC3-II, p62, pro- and mature cathepsin B (CTSB), and cathepsin D (CTSD), in cerebral cortical lysates. Quantification is expressed as mean ± SEM normalized to Actin (*n* = 9). Mature cathepsin B and D were significantly increased in Tg-veh and reduced toward NTg-veh levels by 1H10, whereas the pro-forms of cathepsin B and D showed only non-significant trends across groups. Molecular weight markers (kDa) are indicated to the left of each immunoblot. Statistical analyses were performed using one-way ANOVA followed by Tukey’s post-hoc test unless otherwise indicated. For Congo red plaque quantification, comparisons between Tg-veh and Tg-1H10 mice were analyzed using a two-tailed Student’s t-test. Significance levels are indicated as *p < 0.05, **p < 0.01, ***p < 0.001, NTg-veh versus Tg-veh; ^#^*p* < 0.05, ^###^*p* < 0.001, Tg-veh versus Tg-1H10; ns, not significant

Consistent with previous reports, 5XFAD mice exhibited robust amyloid pathology. The 6E10 antibody, which recognizes the N-terminal region of human Aβ, detected full-length APP (~ 100 kDa) and the β-secretase–derived C-terminal fragment (β-CTF/C99, ~ 16 kDa) on the same blot, together with abundant Congo red–positive plaques. Both APP and β-CTF levels were significantly elevated in Tg-veh mice compared with NTg-veh controls, consistent with enhanced amyloidogenic processing. Chronic administration of 1H10 did not significantly alter APP levels, β-CTF abundance, or amyloid plaque burden, although a modest, non-significant reduction in β-CTF was observed. These findings suggest that 1H10 has little effect on amyloid deposition in this model (Fig. 4A, B).

In contrast, tau pathology was selectively affected by 1H10 treatment. Compared with NTg-veh controls, Tg-veh mice exhibited significant increases in tau phosphorylation at Thr205 and Ser214, accompanied by elevated total tau expression. When normalized to Actin, phosphorylation at both sites was significantly reduced in Tg-1H10 mice relative to Tg-veh mice. Total tau levels were not significantly altered by chronic 1H10 treatment, although a modest decreasing trend was observed (Fig. 4C).

To determine whether these changes were associated with alterations in autophagy–lysosomal pathways, we analyzed markers of autophagy and lysosomal protease processing. Tg-veh mouse brains showed significant accumulation of LC3-II. Chronic 1H10 treatment significantly reduced LC3-II levels when normalized to Actin. Notably, LC3-I levels were also reduced by 1H10 treatment (Fig. 4D). In addition, the mature forms of cathepsins B and D were significantly increased in Tg-veh mice, consistent with altered lysosomal protease homeostasis under pathological conditions associated with impaired lysosomal function [42–46]. Chronic 1H10 treatment reduced the levels of mature cathepsin B and D toward those observed in NTg-veh controls, whereas the proforms showed only non-significant trends across groups (Fig. 4D). In contrast, p62 levels did not differ significantly among NTg-veh, Tg-veh, and Tg-1H10 groups and were not affected by 1H10 treatment (Fig. 4D).

Taken together, these results demonstrate that chronic 1H10 administration attenuates pathological tau phosphorylation in 5XFAD mice without significantly affecting amyloid pathology. These changes are accompanied by partial restoration of lysosomal protease maturation profiles toward non-transgenic levels and reductions in both LC3-I and LC3-II, suggesting modulation of autophagy–lysosomal homeostasis.

## Discussion

In the present study, we show that the small molecule 1H10 acts as a zinc delivery agent that enhances lysosomal function and autophagic flux in cellular models, with in vivo findings consistent with modulation of these pathways, and attenuates tau pathology in the 5XFAD mouse model of Alzheimer’s disease (AD). Chronic administration of 1H10 exerted minimal effects on amyloid deposition but significantly reduced phosphorylated tau levels and was accompanied by modest trends toward cognitive improvement. These findings indicate that 1H10 preferentially engages tau-related clearance pathways rather than directly modulating amyloid pathology.

The mechanistic basis for this effect lies in the zinc-binding properties of 1H10. We confirmed that 1H10 does not bind zinc with excessively high affinity, allowing intracellular zinc levels to increase into a detectable range (≥ 1 µM), as visualized using the Zinpyr-1 dye (K_d_ for zinc, 0.7 nM) [32, 47, 48]. Although the zinc-binding affinity of 1H10 is moderate, it is sufficient to facilitate intracellular zinc mobilization without inducing zinc-mediated toxicity. Excessive intracellular zinc is known to promote ROS generation, lysosomal membrane permeabilization (LMP), and subsequent cell death [49, 50]. In contrast, 1 µM 1H10 achieved lysosomal activation while avoiding these deleterious effects. Physiologically, labile zinc levels are tightly regulated, existing at micromolar concentrations in plasma, nanomolar concentrations in extracellular fluid, and picomolar concentrations in the cytosol [51–54]. Accordingly, direct zinc supplementation is unlikely to achieve controlled intracellular delivery, highlighting the importance of zinc carriers capable of spatially and quantitatively regulated zinc mobilization.

This behavior also helps explain the apparently distinct actions of 1H10 observed across our studies. Because 1H10 is a moderate-affinity, membrane-permeant zinc binder, its biological effect is influenced by the prevailing intracellular zinc environment rather than by an intrinsic chelating or zinc-donating property. The lysosome serves as a major intracellular zinc reservoir and buffering compartment for labile zinc. Consistent with this role, previous studies using the lysosome-targeted probe SpiroZin2 demonstrated that lysosomes rapidly accumulate zinc following extracellular zinc exposure and dynamically regulate intracellular zinc homeostasis [55].

This framework reconciles the different activities of 1H10 observed under distinct experimental conditions. In our previous work supporting the EAE study, cortical neurons were first exposed to supraphysiological zinc concentrations (300 µM Zn^2+^ for 15 min), creating a zinc-overloaded state. Under these conditions, 1H10 (10–40 µM) reduced the elevated pool of intracellular labile zinc and exhibited a functional zinc-chelating effect. In contrast, in the present study, primary cortical neurons were treated with 1 µM 1H10 under basal conditions without exogenous zinc loading. Under these conditions, 1H10 increased lysosomal labile zinc and enhanced lysosomal acidification and proteolytic activity, consistent with a zinc-delivery mechanism. These findings indicate that the biological activity of 1H10 depends on cellular zinc status. When intracellular labile zinc is excessively elevated, 1H10 can reduce the free-zinc pool through zinc binding. Under physiological conditions, however, its predominant effect is to facilitate intracellular zinc redistribution and increase lysosomal zinc availability.

The choice of disease model in the present study followed from this latter activity. Because lysosomal dysfunction is increasingly recognized as a key contributor to AD pathogenesis [1–3], enhancement of lysosomal degradative capacity has emerged as a promising therapeutic strategy. We therefore hypothesized that a small molecule capable of increasing lysosomal zinc availability and promoting lysosomal activity would be beneficial in AD. Importantly, the findings of the present study support the conclusion that, under physiological non-zinc-overloaded conditions, 1H10 acts predominantly as a zinc delivery agent that enhances lysosomal function. Accordingly, the reduction in tau pathology observed in 5XFAD mice is most consistent with improved lysosomal clearance mediated by controlled intracellular zinc delivery rather than zinc chelation.

Zinc has previously been implicated in amyloid plaque formation through extracellular zinc–Aβ interactions that promote Aβ aggregation [56]. However, the primary action of 1H10 is directed toward intracellular zinc mobilization and lysosomal activation. Although 1H10 may modestly influence extracellular zinc availability by facilitating intracellular zinc redistribution, this effect is unlikely to be sufficient to alter established amyloid plaque burden in vivo. Consistent with this interpretation, we observed no significant reduction in amyloid deposition despite clear improvements in lysosomal markers and tau pathology.

Consistent with its zinc-delivery capacity, 1H10 alleviated markers of lysosomal and autophagic dysfunction in 5XFAD mice. Lysosomal impairment is a well-established feature of AD and contributes to the accumulation of aggregation-prone proteins, including hyperphosphorylated tau. In vehicle-treated 5XFAD brains, we observed accumulation of LC3-II and elevated levels of mature cathepsins B and D. Importantly, increased abundance of mature lysosomal proteases does not necessarily reflect enhanced proteolytic activity but may instead indicate impaired lysosomal acidification and reduced protease turnover, leading to accumulation of functionally inefficient mature enzymes. In line with this interpretation, levels of pro-cathepsin B and D were largely unchanged and p62 levels remained stable across experimental groups, suggesting that the primary defect resides downstream of autophagosome formation—most likely at the level of lysosomal acidification or proteolytic efficiency. This stable p62 pattern is also consistent with previous reports in 5XFAD and related amyloid-driven models, in which p62 has been found to be less sensitive than LC3-II as an in vivo marker of autophagic dysfunction [57–60]. The reasons why p62 fails to accumulate despite lysosomal dysfunction in these models remain unclear; however, the consistent observation of LC3-II elevation without parallel p62 accumulation across multiple amyloid-driven mouse models suggests this reflects a model-specific feature rather than an artifact, and supports the use of LC3-II as the more reliable marker of autophagic flux impairment in the 5XFAD context. Notably, these abnormalities were significantly attenuated by 1H10 treatment, with normalization of LC3-II levels and restoration of mature cathepsin abundance toward NTg-veh levels, collectively suggesting partial normalization of lysosomal homeostasis and degradative capacity in vivo. This interpretation also reconciles the apparently opposite directions of the in vitro and in vivo cathepsin data. In primary neurons with intact lysosomal acidification, 1H10 acutely increased in situ cathepsin B activity and bulk proteolytic activity (Fig. 1D, E), reflecting enhanced enzymatic function of the existing lysosomal protease pool following intralysosomal zinc elevation. This represents an activity-based readout. In the 5XFAD cortex, by contrast, the elevated steady-state abundance of mature cathepsins likely reflects impaired lysosomal protease turnover under conditions of defective acidification, whereas its partial normalization by 1H10 is consistent with restoration of lysosomal turnover rather than suppression of protease expression. Thus, the two observations assess distinct parameters—enzymatic activity versus protein abundance—and are mechanistically complementary rather than contradictory.

These in vivo findings are supported by our cellular data showing that 1H10 reverses bafilomycin A1–induced autophagic blockade and promotes the clearance of overexpressed Aβ and mutant tau proteins. In the cortex of 5XFAD mice, 1H10 selectively reduced phospho-tau levels at Thr205 and Ser214 without significantly affecting total tau expression, indicating preferential removal of pathological tau species rather than global suppression of tau production. Although amyloid plaque burden and APP expression were not altered, the selective attenuation of tau pathology remains noteworthy, given the established association between tau pathology and disease progression in AD [61–63]. Importantly, despite the absence of a measurable reduction in amyloid plaque burden, 1H10 produced a clear attenuation of pathological tau phosphorylation, indicating that modulation of intracellular degradative pathways can yield disease-relevant benefits independently of amyloid clearance. Although 5XFAD mice are primarily an amyloid-driven model, accumulating evidence indicates that tau hyperphosphorylation can arise downstream of amyloid pathology and can be modulated independently of plaque burden, particularly through pathways regulating proteostasis and lysosomal function [64, 65]. This selectivity for pathological phospho-tau over Aβ and β-CTF is consistent with a mechanism based on intracellular lysosomal–autophagic activation and likely reflects a difference in substrate accessibility rather than selective enzymatic discrimination. Pathological phospho-tau is an intracellular substrate directly accessible to the autophagy–lysosomal pathway, whereas most amyloid burden in 5XFAD mice resides in extracellular fibrillar plaques that are inaccessible to intracellular degradative machinery. Likewise, β-CTF is continuously regenerated through constitutive processing of the overexpressed mutant APP transgene. The observed reduction in phospho-tau without corresponding changes in Aβ or β-CTF therefore reflects the differential accessibility of these pathological substrates to lysosomal clearance.

At the behavioral level, 1H10-treated 5XFAD mice exhibited subtle but consistent trends toward improved spatial learning during the acquisition phase of the Morris water maze. Although these improvements did not reach statistical significance, Tg-1H10 mice consistently exhibited intermediate performance between NTg and Tg-veh groups, suggesting a modest shift toward improved spatial learning rather than a full behavioral rescue. These findings align with previous reports showing that enhancement of lysosomal function can yield modest but reproducible behavioral benefits in neurodegenerative disease models [3, 66, 67].

Several factors may account for the limited magnitude of behavioral improvement observed in this study. First, a single dose regimen (30 µg/kg) was employed without prior pharmacokinetic or blood–brain barrier permeability analyses, leaving uncertainty regarding optimal dosing and central drug exposure. Second, treatment was initiated at 3 months of age, when amyloid pathology is already developing in 5XFAD mice. Earlier intervention might be required to influence amyloid accumulation and produce more robust cognitive outcomes. Future studies should therefore examine earlier therapeutic windows, broader dosing regimens, and more detailed regional analyses of Aβ deposition to determine whether 1H10 can influence amyloid pathology at earlier stages of disease progression. Nonetheless, the biochemical and pathological improvements observed suggest that 1H10 achieves sufficient brain exposure and target engagement to modulate lysosomal function.

Although our data support zinc-dependent enhancement of lysosomal proteolytic function, the precise upstream regulatory pathways remain to be fully defined. Future studies examining the involvement of lysosomal biogenesis regulators such as TFEB activation, as well as direct assessment of lysosomal pH dynamics, will be valuable to further delineate the molecular mechanisms underlying 1H10-mediated lysosomal modulation.

Several limitations of the present study should be acknowledged. First, neither Aβ nor tau overexpression induced detectable lysosomal membrane permeabilization in our cellular model, as assessed by galectin-3 puncta formation. We therefore used bafilomycin A1 as a defined pharmacological model of lysosomal dysfunction. Whether 1H10 can mitigate lysosomal damage directly induced by pathological Aβ or tau aggregates remains an important question for future investigation. Second, a dedicated non-transgenic group receiving 1H10 was not included in the chronic treatment cohort. Consequently, the systemic tolerability of long-term 1H10 administration in non-transgenic animals remains to be fully evaluated. Although body weight did not differ between vehicle- and 1H10-treated 5XFAD mice and no systemic toxicity was observed in our previous in vivo study, dedicated safety and toxicology studies will be required before translational development. Third, the in vivo mechanistic evidence presented here is based on convergent steady-state protein markers, including LC3-II, mature cathepsins B and D, and phospho-tau, rather than direct functional measurements in brain tissue. Although we detected an acute increase in intracellular zinc following 1H10 treatment in cultured neurons, the same approach is not informative in chronically treated animals because transient changes in labile zinc are expected to have resolved long before tissue collection. Moreover, established zinc-imaging probes such as TSQ and Newport Green reliably detect only supraphysiological zinc accumulation associated with cytotoxicity and are not sufficiently sensitive to measure the modest physiological-range zinc elevation produced by 1H10 [52, 68–70]. Direct assessment of lysosomal pH in brain tissue is also technically challenging because intact lysosomes are not preserved during tissue processing. In contrast, measurement of cathepsin activity in tissue has been successfully reported by other groups and may provide a valuable complementary readout of lysosomal function [71–74]. Direct confirmation of cathepsin activity would have provided stronger support for the interpretation that the reduction in mature cathepsin B and D abundance reflects restoration of lysosomal function. Nevertheless, the convergence of changes across multiple independent lysosomal and pathological markers supports this interpretation. Future studies incorporating direct measurements of cathepsin activity will be important to further strengthen the mechanistic evidence underlying 1H10-mediated lysosomal activation.

Finally, this study contributes to a more nuanced understanding of zinc biology in the central nervous system. While zinc dyshomeostasis is frequently associated with neurotoxicity and proteostasis failure, our results support the concept that spatially and quantitatively controlled zinc delivery—particularly to lysosomes—may contribute to restoration of degradative capacity and exert neuroprotective effects. In this regard, 1H10 represents a potential prototype for lysosome-targeted zinc modulators, and further optimization of its pharmacokinetic and pharmacodynamic properties may enable the development of next-generation therapeutics for neurodegenerative diseases characterized by impaired proteostasis.

## Supplementary Information

Below is the link to the electronic supplementary material.

Supplementary Material 1

## Data Availability

The datasets generated and/or analyzed during the current study are available from the corresponding author upon reasonable request.

## Abbreviations

AD: Alzheimer’s disease
AMPK: AMP-activated protein kinase
Aβ: Amyloid-β
APP: Amyloid precursor protein
BafA1: Bafilomycin A1
CTSB: Cathepsin B
CTSD: Cathepsin D
EAE: Experimental autoimmune encephalomyelitis
LMP: Lysosomal membrane permeabilization
LTR: LysoTracker Red
NTg: Non-transgenic littermates
MWM: Morris water maze
pMCAO: Permanent middle cerebral artery occlusion
TFEB: Transcription factor EB
Tg: Transgenic
NTg-veh: Vehicle-treated non-transgenic mice
Tg-veh: vehicle-treated 5XFAD transgenic mice
Tg-1H10: 1H10-treated 5XFAD transgenic mice
Zinpyr-1: ZP-1
